# qPCR-Based Monitoring of 2-Methylisoborneol/Geosmin-Producing Cyanobacteria in Drinking Water Reservoirs in South Korea

**DOI:** 10.3390/microorganisms11092332

**Published:** 2023-09-16

**Authors:** Jung Eun Lee, Rumi Park, Mina Yu, Myeongseop Byeon, Taegu Kang

**Affiliations:** Han River Environment Research Center, National Institute of Environmental Research, 819 Yangsoo-ri, Yangpyeong-goon, Incheon 12585, Gyeonggi-do, Republic of Korea; prm3270@korea.kr (R.P.); zmffjq2435@korea.kr (M.Y.); zacco@korea.kr (M.B.); taegu98@korea.kr (T.K.)

**Keywords:** geosmin, 2-MIB, *mic* gene, *geo* gene, drinking water reservoirs, cyanobacteria, quantitative PCR (qPCR)

## Abstract

Cyanobacteria can exist in water resources and produce odorants. 2-Methylisoborneol (2-MIB) and geosmin are the main odorant compounds affecting the drinking water quality in reservoirs. In this study, encoding genes 2-MIB (*mic*, *monoterpene cyclase*) and geosmin (*geo*, *putative geosmin synthase*) were investigated using newly developed primers for quantitative PCR (qPCR). Gene copy numbers were compared to 2-MIB/geosmin concentrations and cyanobacterial cell abundance. Samples were collected between July and October 2020, from four drinking water sites in South Korea. The results showed similar trends in three parameters, although the changes in the 2-MIB/geosmin concentrations followed the changes in the *mic*/*geo* copy numbers more closely than the cyanobacterial cell abundances. The number of odorant gene copies decreased from upstream to downstream. Regression analysis revealed a strong positive linear correlation between gene copy number and odorant concentration for *mic* (*R*^2^ = 0.8478) and *geo* (*R*^2^ = 0.601). In the analysis of several environmental parameters, only water temperature was positively correlated with both *mic* and *geo*. Our results demonstrated the feasibility of monitoring 2-MIB/geosmin occurrence using qPCR of their respective synthase genes. Odorant-producing, gene-based qPCR monitoring studies may contribute to improving drinking water quality management.

## 1. Introduction

Many microorganisms, including cyanobacteria, actinomycetes, and certain fungi, produce malodorous compounds [1]. In water reservoirs, several species of cyanobacteria produce earthy- or musty-smelling compounds as secondary metabolites [2,3,4]. The main cyanobacterial odorants affecting the quality of drinking water in reservoirs are 2-methylisoborneol (2-MIB) and geosmin [5,6]. The odor threshold value for both is <10 ng L^−1^, but their presence is often observed at 5 ng L^−1^ [7]. Although neither 2-MIB nor geosmin poses a serious health threat to humans or aquatic animals, the perception of drinking water has raised suspicions among consumers about drinking water safety and led to complaints [8]. Worldwide, reports on cyanobacteria associated with 2-MIB and geosmin in drinking water have gradually increased over the last three decades, with more than 30 reports each [9,10].

Paldang Lake, fed by the Han River, is the largest (storage capacity of ~244 × 10^6^ tons) drinking water reservoir in South Korea [11]. It is located near the capital city of Seoul and serves as the principal water source for more than 25 million people (50% of the population). In this reservoir, the drinking water monitoring standard for odorant compounds is 20 ng L^−1^, and analyses of cyanobacterial numbers and unpleasant malodorous compounds are conducted once weekly, as mandated by the Korean Ministry of Environment in South Korea in 1998. The first report of a cyanobacterial bloom occurred in 2012 when the concentration of geosmin was high; in the fall of 2018, the occurrence of 2-MIB was also high [12]. Since then, algal bloom events have occurred several times, resulting in an increase in the number of earthy/musty odor complaints in the drinking water from Paldang Lake [13]. These odorant events mainly occurred upstream of the North Han River and Paldang Lake. 2-MIB and geosmin are difficult to remove because they are extremely stable and slowly degrade naturally in a water environment or by boiling. In addition, conventional drinking water treatment methods such as chlorine, ozone, coagulation–sedimentation, and rapid sand filtration are ineffective in removing these substances [4,14,15,16]. Therefore, an accurate early analysis of 2-MIB/geosmin-producing cyanobacteria would contribute to better management of drinking water resources.

Cyanobacterial species, including those responsible for odorant production, are typically classified and enumerated using microscopy, and odorant concentrations such as of 2-MIB/geosmin are determined using gas chromatography (GC). However, for monitoring purposes, microscopy-based methods are time-consuming and require specially trained personnel because it is difficult to distinguish between odorant producers and non-producers [17]. While GC can provide accurate quantification of odorants in water, it offers no information about their producers at the source and requires specialized equipment [5]. The discovery of the 2-MIB and geosmin synthase genes *mic* and *geo* have enabled the development of a quantitative PCR (qPCR) approach to rapidly detect even low gene copy numbers and enable early on-site detection of the responsible cyanobacteria [8,18].

This approach requires specific primers that are well-designed based on *mic* and *geo* sequence information and are applicable to fielding samples from various geographical locations [9]. There may be regional differences within a single species of cyanobacteria. Therefore, it is necessary to establish a qPCR-based analytical method. In addition, because several cyanobacteria can exist in water resources and produce odorants (such as 2-MIB and geosmin), it is necessary to analyze and characterize odorant-producing species simultaneously. However, field studies on 2-MIB/geosmin-producing cyanobacteria using qPCR are lacking in Asia, including Korea, and the existing applications are aimed at detecting only one odorant producer. 

Therefore, we developed a molecular method to quantify the 2-MIB and geosmin synthase genes and evaluated the utility of a qPCR-based approach for odorant monitoring in drinking water reservoirs.

## 2. Materials and Methods

### 2.1. Monitoring Sites and Water Sampling

The Han River comprises two large tributaries (the North and South Han Rivers), covers an area of 26,000 km^2^, and is 5417 km long [19]. The North Han River flows into Paldang Lake, where there are major upstream and downstream dams. The last dam before the mainstream is the Paldang Dam, which is the main drinking water source for the region. In recent years, complaints regarding odorous compounds have increased near the North Han River, and high concentrations of odorous compounds have been reported [20].

**Figure 1:**
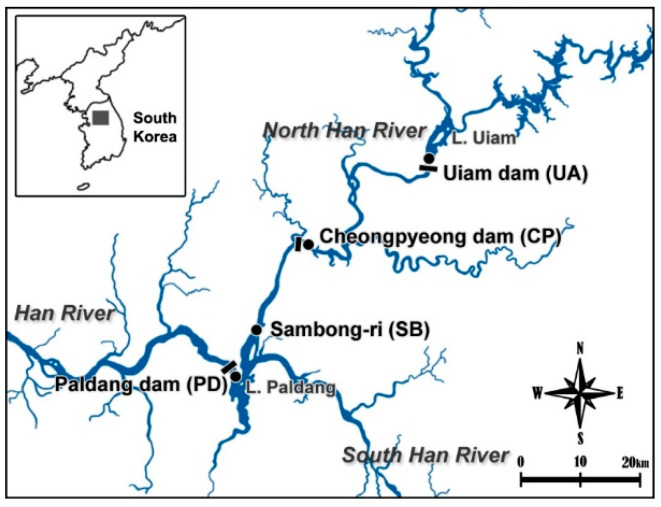
Water sampling locations in the North Han River Basin, South Korea.

Samples for odorant monitoring were obtained from Paldang Lake and the North Han River in South Korea. We investigated these field sites, including Uiam Dam (UA; N 37°50′15.02″, E 127°40′33.22″), located upstream of the North Han River; Cheongpyung Dam (CP; N 37°43′27″, E 127°25′33″); Sambong-ri (SB; N 37°35′39.21″, E 127°20′28.7″); and Paldang Dam (PD; N 37°31′17.02″, E 127°17′00.02″) in Paldang Lake (Figure 1). Water samples were taken at the sampling sites at a depth of 0.5 m each using an 8 L water sampler (Wildco, Jacksonville, FL, USA) [21]. Water samples for analyzing water quality and odorous compounds were placed in 2 L plastic bottles and 50 mL glass bottles, respectively, and samples for the analysis of *mic* and *geo* using quantitative PCR (qPCR) were collected in 1 L sterile plastic bottles. The samples were stored under cold and dark conditions during transportation to the laboratory [22,23].

### 2.2. Isolation and Enumeration of Cyanobacteria

The water samples collected for the cyanobacterial analyses were placed in 500 mL plastic bottles and fixed immediately with Lugol’s iodine solution (final concentration: 2% *w*/*v*). To quantify cyanobacteria, 1 mL fixed specimens were placed in a Sedgwick–Rafter counting chamber and allowed to settle for at least 15 min, followed by observation under a phase microscope (Eclipse 80i; Nikon Corp., Sendai, Japan). The number of cells per unit area was observed at 100–1000× magnification, and the total concentration was calculated. Unialgal strains were isolated using the Pasteur capillary pipette method [24]. Single filaments were selected using a Pasteur capillary pipette under a dissecting microscope (Nikon, Tokyo, Japan). The isolates were then placed in a 24-well plate containing a liquid BG-11 medium and cultured at 25 °C under a 12 h:12 h dark/light cycle (40 μmol/m^2^/s) for gas chromatography–mass spectrometry (GC-MS) and molecular characterization.

### 2.3. Primer Design for Real-Time qPCR

Odorant-producing cyanobacteria were isolated from Paldang Lake, and pure cultures were obtained. The odorant synthase gene sequences were deposited with GenBank (accession numbers MT360266, MT515744, and ON365769–ON365771). The odorant gene sequences from the isolated odorous cyanobacteria (*Pseudanabaena* sp. and *Anabaena* sp.) and NCBI databases were used to establish the primer design for the real-time qPCR amplification of *mic* and *geo* using the SYBR Green system. Regions with high detectability were selected by aligning the isolated and referred sequences using BioEdit software 7.2 and were used to design the two primer sets (Table 1).

Two 2-methylisoborneol (2-MIB) primers (3909F/4028R) were designed to identify *mic*, which encodes monoterpene cyclase, the enzyme that cyclizes 2-methyl GPP to 2-MIB [25,26], and specifically, the region that encodes the highly conserved Mg^2+^-binding site. The target DNA fragment for the detection of geosmin, *geo*, is included in *gsy1*, a putative geosmin synthase sequence [27] that is part of the geosmin synthesis operon of *Anabaena ucrainica*. The G-C content (%) of the selected regions and the potential for secondary structure formation were confirmed using OligoCalc (biotools.nubic.nothwastern.edu/Oligocalc.html accessed on 12 September 2023).

### 2.4. Quantification of 2-MIB and Geosmin Synthase Genes with qPCR

Quantifications of *mic* and *geo* were performed using a 7500 real-time PCR instrument (Applied Biosystems, Thermo Fisher Scientific, Boston, MA, USA) with 7500 software v2.3. SYBR Green was used as a fluorescent intercalator for double-stranded DNA in the real-time PCR analysis. The PCR was performed in a 20 µL volume containing 3 µL of template DNA, 1 µL (10 pM) of specific primers, 10 µL of PowerUp^TM^ SYBR^TM^ Green Master Mix (Applied Biosystems, Boston, MA, USA), and 5 µL of sterile deionized water. The PCR conditions for both the *mic* and *geo* reactions were pre-incubation at 95 °C for 10 min followed by 40 cycles of 15 s of denaturation at 95 °C for 30 s and annealing/extension at 59 °C. Next, the temperature was raised from 60 °C to 95 °C, and a melting temperature analysis of the PCR products was performed to confirm the specificity of the primers.

As standards for *mic* and *geo* quantification, plasmids carrying the target fragments for the 3909F/4028R and 2014F/2163R primers of the *Pseudanabaena* sp. and *Anabaena* sp., respectively, were generated using TOPcloner^TM^ TA (Enzynomics, Daejeon, Republic of Korea). Standard curves were prepared using tenfold serial dilutions of the cloned plasmid (eight dilutions) and included in each qPCR analysis. The quantification of *mic* and *geo* in the samples used an external standard method based on standard curves. The copy numbers of the standards were calculated as follows [28]:

$$N\left(copies/mL\right)=\frac{C_{DNA}(g/\mu L)\times A_{N}(bp/mole)\times 10^{3}}{L_{plasmid}(bp)\times {MW}_{bp}(g/mole)}$$

where C_DNA_ is the DNA concentration (g/µL) detected using a NanoDrop One spectrophotometer (Thermo Fisher Scientific, Boston, MA, USA) in the standard solution, A_N_ is Avogadro’s number (bp/mole), L_plasmid_ is the lengths (bp) of the plasmid-inserted 2-MIB and geosmin synthase genes (3947 and 3957 bp, respectively), and MW_bp_ is the molecular weight of the base pair (660 g/mol).

### 2.5. Quantification of 2-MIB and Geosmin Synthase Genes Using qPCR

Cyanobacteria were concentrated by filtering 100–300 mL of collected water through a membrane filter (Merck Millipore, Molsheim, France) with a 0.45 µm pore size. DNA was extracted from the filter using a Dneasy PowerWater Kit (Qiagen, Solingen, Germany) according to the manufacturer’s instructions. The concentrated water filters were inserted into a bead tube, and the cells were lysed with bead-beating using a vortex and heated in a water bath at 65 °C for 10 min, after which DNA was extracted using 30 µL of EB (elution buffer) solution according to the kit manual. All extracted DNA samples were stored at −70 °C until qPCR analysis.

### 2.6. Odorant Analysis

The concentrations of 2-MIB and geosmin in the field samples were measured using headspace solid-phase microextraction (HS-SPME) coupled with GC-MS (Agilent 5977 B, Agilent Technologies, Santa Clara, CA, USA). A 10 mL sample containing 3 g of added NaCl was adsorbed onto the activated SPME fiber (50/30 µm of DVB/CAR/PDMS; Supelco, Sigma-Aldrich Corp., St. Louis, MO, USA) for 30 min at 70 °C, with stirring at 400 rpm. The target component of the fiber was desorbed at 270 °C for 4 min and injected into a GC-MS DB-5MS column for analysis. A mixed standard solution (47525-U; Supelco Inc., Bellofonte, PA, USA) for the quantification of the geosmin and 2-MIB in the samples was simultaneously analyzed in the sample run and quantified according to the external standard method.

### 2.7. Environmental Parameter Analysis

At each sampling site, the water temperature (WT) and dissolved oxygen (DO) were measured using a multiparameter water quality checker (YSI EXO; YSI Inc., Yellow Springs, OH, USA). Water quality parameters were analyzed using standard South Korean methods [29]. Chlorophyll a concentration (Chl-a) was measured using a UV-Vis spectrophotometer (Agilent Carry 3500; Agilent, Santa Clara, CA, USA) [30]. Both NH_3_-N and PO_4_-P were analyzed with continuous-flow methods using a continuous-flow analyzer (CFA; Seal Analytical AAC, Seal, WI, USA).

### 2.8. Statistical Analysis 

The relationships between the 2-MIB/geosmin gene copy numbers, 2-MIB/geosmin concentrations, and cyanobacterial cell numbers were evaluated using regression analysis using SigmaPlot ver.10.0 (Systat Software Inc., San Jose, CA, USA). Significance was determined using Student’s t-test, with *p* < 0.05 considered statistically significant. The correlation between the 2-MIB/geosmin gene copy numbers and environmental factors was also evaluated using Student’s t-test. The Pearson correlation coefficient (R) was used as an indicator of linear relationships (SPSS ver.20; IBM Corp., Armonk, NY, USA).

## 3. Results and Discussion

### 3.1. Standard Curves for Real-Time qPCR

The primers used for quantitative PCR (qPCR) were designed from the odorant gene sequences deposited with GenBank after the pure isolation of the odorant-producing cyanobacteria from Paldang Lake and the sequences in the NCBI database (Table 1). After isolation, pure cultures were obtained. Sequences of the odorant synthase genes were deposited with GenBank (accession numbers: MT360266, MT515744, and ON365769–ON365771). The odorous gene sequences from the isolated cyanobacterial sequences (*Pseudanabaena* sp. and *Anabaena* sp.) and NCBI databases were used to establish the primer design for a real-time qPCR assay of *mic* and *geo* using the SYBR Green System (Table 1). To prepare the standards for gene quantification, DNA extracted from the *Pseudanabaena* sp. (#ON365771) and Anabaena crassa (#ON365770) was used. The extracted DNA was PCR-amplified using the designed primers, and the PCR products were cloned using TOPcloner^TM^ TA (Enzynomics). After cloning, the molecular weight of the plasmid was determined, the copy number was calculated, and tenfold serial dilutions were prepared (10–10^7^ copies/µL) and quantified using real-time qPCR. 

**Figure 2:**
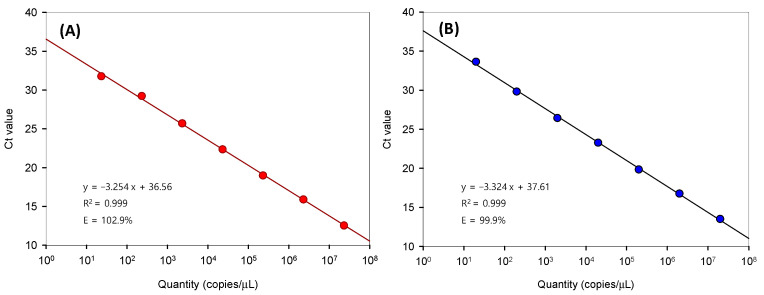
Standard curves for the real-time qPCR-based analysis of (**A**) *mic* and (**B**) *geo* based on data obtained using SYBR Green real-time qPCR.

The two standard curves (Figure 2) demonstrated strong linearity with high correlation coefficients (R^2^ = 0.9999). The efficiency (E) of the real-time qPCR was calculated as E = (10-^1/S^ –1), where S is the slope of the standard curve [8,17]. The E values for *mic* and *geo* were 102.9% and 99.9%, respectively (Figure 2). These high correlation coefficients and efficiencies indicated that the qPCRs using these standard curves were reasonable and that the detection limit of this qPCR analysis was <10 copies/mL.

### 3.2. Application of qPCR-Based Monitoring in Drinking Water Reservoirs

Our real-time qPCR method was used to quantitatively monitor 2-MIB- and geosmin-producing cyanobacteria in the North Han River, which is the primary drinking water reservoir in South Korea. Water samples were collected in 2020 from three upstream sites, Uiam Dam (UA), Cheongpyung Dam (CP), and Sambong-ri (SB), and one downstream site, Paldang Dam (PD), between July and October, corresponding to a period of high-level cyanobacterial growth. In addition to the copy number of odorous-producing genes determined with the qPCR analysis, water samples were analyzed for 2-MIB and geosmin concentrations (GC-MS) and cyanobacterial cell abundance (microscopy). 

**Figure 3:**
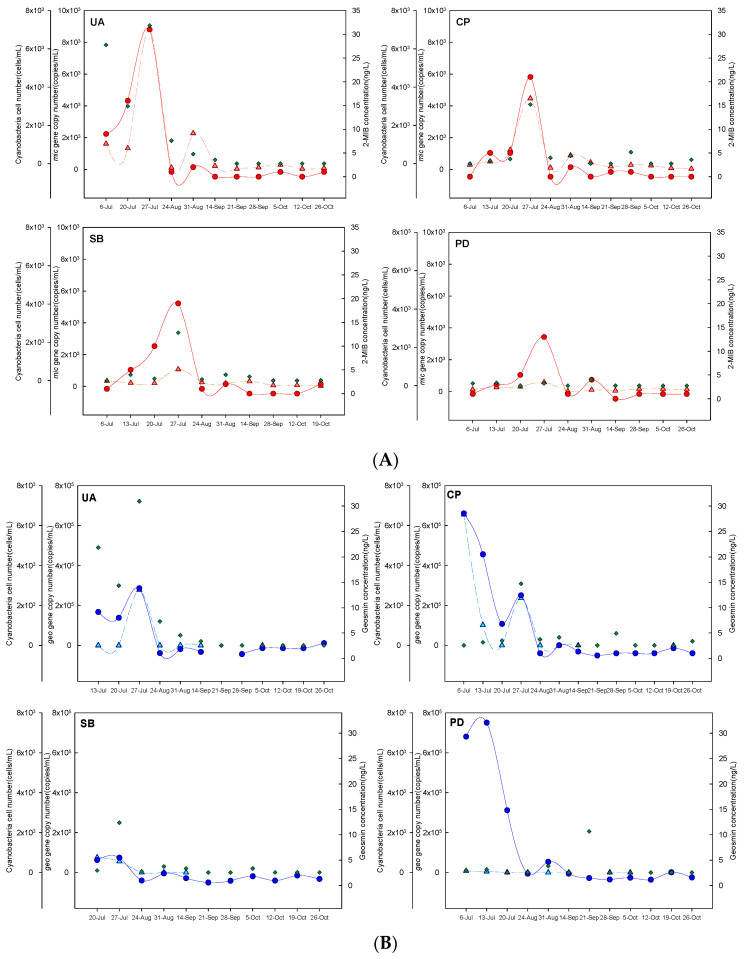
Time course of (**A**) *mic* copy number, 2-MIB concentration, and cyanobacterial cell abundance and (**B**) *geo* copy number, geosmin concentration, and cyanobacterial cell abundance at the four sampling sites. ▲, *mic* or *geo* copy number as determined with qPCR; ●, 2-MIB or geosmin concentration as determined with GC-MS; and ◆, cyanobacterial cell abundance as determined with microscopy.

The three parameters, *mic* copy number, 2-MIB concentration, and cyanobacterial cell abundance, followed similar trends (Figure 3A). The trends in 2-MIB concentration and *mic* copy number were nearly identical, and the overall change in the 2-MIB concentration followed the change in the *mic* copy number more closely than the change in cell abundance. A previous study of three reservoir sites showed that trends in *mic* abundance closely followed the total 2-MIB concentration in many but not all samples [17]. The trends in 2-MIB concentration and cyanobacterial cell abundance were also roughly similar; however, at several sampling times within the UA and PD sites, both the *mic* copy number and the 2-MIB concentration were low, whereas the cyanobacterial cell abundance was high. In particular, at the PD site, when the 2-MIB concentration and *mic* copy number were low and the cyanobacterial cell abundance was high, cyanobacterial species that did not produce 2-MIB were dominant (Table S1).

Both *mic* copy number and 2-MIB concentration increased gradually, beginning in June, at all sites, peaked on 27 July, and decreased thereafter. This pattern is consistent with the increased growth of 2-MIB-producing cyanobacteria during the warm season (in South Korea, from July to October) [31]. In a previous study, the 2-MIB concentration was highest in July, as determined over a nearly 3-year period [31]. A study of the time courses of *mic* copy number and 2-MIB concentration showed that both were highest in July and August [17]. At all four sampling sites, the *mic* copy numbers and 2-MIB concentrations were the highest in June and July, ranging from 1 × 10^1^ to 9 × 10^3^ copies mL^−1^ and 2 to 31 ng L^−1^, respectively. 

Gene analysis of *mic* in aquatic systems in other regions showed that it was detected in most samples, with amounts ranging from 10^3^ to 10^6^ copies L^−1^ and 2-MIB concentrations from 7.2 to 45.3 ng L^−1^, indicating higher gene and 2-MIB concentrations in drinking water than those observed in this study [32]. Another study of drinking water showed lower 2-MIB concentrations ranging from 2.39 to 7.32 ng L^−1^ [33]. 

Figure 3B shows the time course of the *geo* copy number as determined with qPCR, the geosmin concentration measured with GC-MS, and the cyanobacteria cell abundance determined with microscopy. Similar to with 2-MIB, all three parameters followed a similar pattern during the study period, with the *geo* copy number and geosmin concentration tending to be higher than the cyanobacterial cell abundance. Tsao et al. [8] investigated the geosmin concentration and *geo* copy number in the Miponga Reservoir, South Australia, and found that the two parameters were closely related, with *geo* copy numbers ranging from 10^2^ to 10^5^ mL^−1^, similarly to our results.

Notably, the *geo* and cell counts were lower at the PD site, and the geosmin concentrations, as well as the total number of cyanobacteria, were higher in a period dominated by *Anabaena circinalis* (Table S1), which is known to be a major producer of geosmin [8]. At all sites, *geo* copy number and geosmin concentrations increased from June to July and then decreased, with values ranging from 3 to 6.5 × 10^5^ copies mL^−1^ and 5 to 32 ng L^−1^, respectively. These results are similar to those of Harris et al. [34], who found that the geosmin concentrations and cyanobacterial cell abundance in drinking water reservoirs over a 14-year period were highest in June and July.

The total numbers of cyanobacterial cells, dominant species of cyanobacteria, cell abundances, and odorant concentrations are summarized in Table S1. Cyanobacteria are the most major producers of odorants, which several species have been reported to secrete. Multiple morphologically similar species or genera can coexist in water sources, but not all will have the same potential to synthesize odorants [4]. Therefore, 2-MIB/geosmin concentrations and cell abundance counts can be used to determine which species are potential odorant producers within an overall cyanobacterial population. The total number of cyanobacterial cells varied from 10 to 7221 cells mL^−1^, and the dominant species were the *Pseudanabaena* sp. (10–4900 cells mL^−1^), the *Phormidium* sp. (1600–2100 cells mL^−1^), the *Microcystis* sp. (60 cells mL^−1^), the *Anabaena* sp. (90–120 cells mL^−1^), and the *Merismopedia* sp. (20–1250 cells mL^−1^). The *Pseudanabaena* sp. was dominant in most cases at UA, CP, and SB, which are upstream of the North Han River, whereas the *Anabaena* sp. was dominant at the PD site downstream. When the *Pseudanabaena* sp. was dominant, the cell number exceeded 3000 cells mL^−1^, the 2-MIB concentration was high (16–31 ng L^−1^), and geosmin was detected at a relatively low concentration. The *Phormidium* sp. was dominant on July 27 at the CP and SB sites, with a 2-MIB concentration of approximately 20 ng L^−1^. The *Anabaena* sp. was dominant at the PD site, and the geosmin concentrations were as high as 29 ng L^−1^ and 32 ng L^−1^, whereas 2-MIB was hardly detected. The *Pseudanabaena* sp. is a major producer of 2-MIB in many countries [35,36] and was confirmed to be the main cause of 2-MIB production in South Korea in 2018 [12]. In this study, the dominant species of cyanobacteria was the *Pseudanabaena* sp., and the 2-MIB concentration was high.

**Figure 4:**
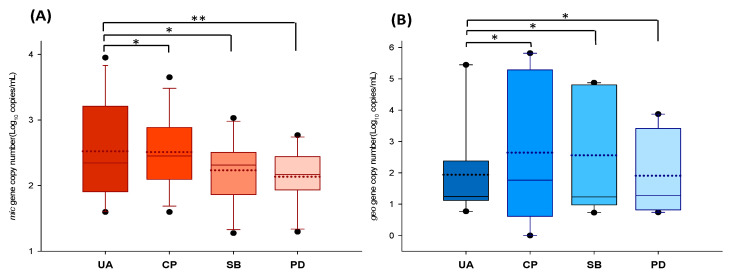
Comparison of (**A**) *mic* and (**B**) *geo* copy numbers at the four sampling locations. The horizontal dotted line within each box represents the mean value. Error bars represent standard deviation. The circles indicate the 5th and 95th percentiles (Student’s *t*-test; * *p* < 0.5, ** *p* < 0.05).

Figure 4 shows the ranges of the *mic* and *geo* copy numbers determined at the sampling sites during the study period. The UA site, the most upstream of the study monitoring points, has previously been reported to have high odorant concentrations [20] and was used as a control point to determine how these changed as the odorants moved downstream. For both genes, the copy numbers differed among the four sampling locations and decreased from upstream to downstream. In the t-tests, the copy numbers of the odorous genes were significantly different (*p* < 0.5 and *p* < 0.05) depending on their location in the stream. For *mic*, the copy number was highest (log1.59–log3.95 copies mL^−1^) at the most upstream site (UA) and progressively decreased downstream, such that the lowest copy number (log 1.29–log 2.48 copies mL^−1^) was detected at PD (Figure 4A). A similar pattern was observed for *geo* (Figure 4B), with the highest gene copy number (up to log 5.82 copies mL^−1^) detected at the upstream sites UA and CP and decreasing downstream.

The Han River has two main branches, the South Han and North Han Rivers, which merge at Paldang Dam. In a study of water quality trends, the values of the most measured parameters have decreased from the upper stream to the lower stream of the Han River [37]. Other geosmin-monitoring studies have also reported decreasing concentrations from upstream to downstream sites [38]. Thus, at the Paldang Reservoir, the most downstream sampling site in this study, the concentrations of many indicators of water quality may have been diluted due to the increased inflow resulting from the confluence of the two river branches.

### 3.3. Regression Analysis of Gene Copy Numbers, Odorant Concentrations, and Cyanobacterial Cell Abundance

**Figure 5:**
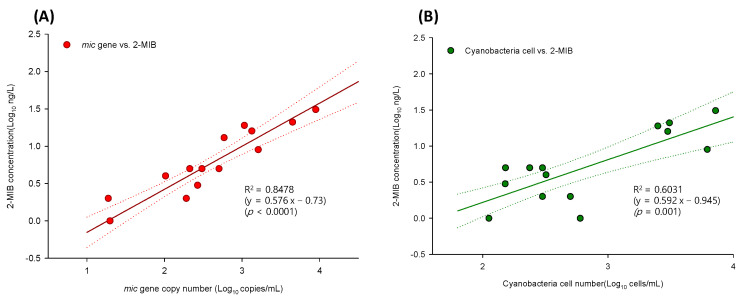

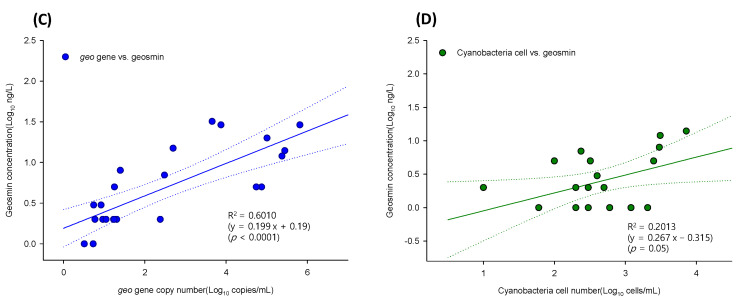
Relationships between *mic* copy number and 2-MIB concentration (**A**), cyanobacterial cell abundance and 2-MIB concentration (**B**), *geo* copy number and geosmin concentration (**C**), and cyanobacterial cell abundance and geosmin concentration (**D**), with 95% confidence intervals (dotted lines).

The relationships among gene copy number, 2-MIB/geosmin concentration, and cyanobacterial cell abundance were evaluated. Figure 5A shows the results of the log–log model and regression analysis of *mic* copy number and 2-MIB concentration. A positive linear correlation (R^2^ = 0.8478, *p* < 0.0001) was determined from the equation y = 0.576x − 0.73 (Figure 5A) and indicated that one *mic* copy corresponds to 4–50 fg of 2-MIB. A previous study [32] examined the correlation between *mic* copy number and 2-MIB concentration and found a log-linearity coefficient of 0.604 2-MIB (ng L^−1^) per *mic* copy in field samples, with 2-MIB production ranging from 11 to 57 fg per *mic* copy. Our microscopic analysis of 2-MIB concentration and cyanobacterial cell abundance (Figure 5B) showed a positive linear correlation between the two. However, as reflected by the correlation coefficient, R^2^ = 0.6031, the correlation was weaker than that between 2-MIB concentration and *mic* copy number.

Figure 5C shows the positive relationship between *geo* copy number and geosmin concentration (R^2^ = 0.601, *p* < 0.001), determined with the equation y = 0.199x + 0.19. According to the log–log regression analysis, the estimated geosmin production was 1–186 fg per *geo* copy. Su et al. [39] reported a geosmin production potential of 1–40 fg of geosmin per *geo* copy, whereas another study reported an average geosmin production of ~100 fg cell^−1^ [40]. Regression analysis of *geo* copy number and geosmin concentration also showed that the distribution was wider than that of *mic*. A previous study reported a significant correlation between geosmin synthase gene copy number and geosmin concentration (R^2^ = 0.694, *p* < 0.001), with field samples showing a broad distribution pattern [39]. 

A comparison of cyanobacterial cell abundance and geosmin concentration (Figure 5D) showed a weak correlation (R^2^ = 0.2013, *p* = 0.05). Similarly, another study found no correlation between cell density and *geo* expression levels, as determined with qPCR [41]. The geosmin-producing cyanobacterial genus *Anabaena* was reported to be responsible for 46% of geosmin events [42], and the geosmin concentration was strongly correlated with *Anabaena* cell density (R^2^ = 0.97) [8]. In this study, the total cyanobacterial cell abundance and thus geosmin-producing and non-producing species were evaluated, explaining the weaker correlation between cyanobacterial cell abundance and geosmin concentration determined in this analysis.

### 3.4. Environmental Factors

**Figure 6:**
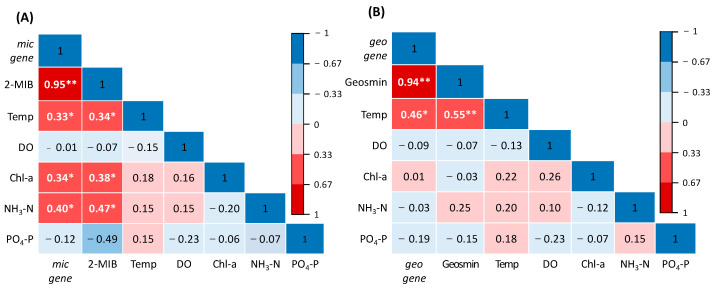
Relationship matrix of the Pearson correlation coefficients (R) for *mic* (**A**) and *geo* (**B**) and environmental factors (* *p* < 0.05, ** *p* < 0.01).

The odorant concentrations and gene copy numbers were compared to the levels of several environmental factors, including water temperature, dissolved oxygen, Chl-a, NH_3_-N, and PO_4_-P, using correlation analysis. As shown in the correlation matrix in Figure 6A, *mic* was positively correlated with 2-MIB concentration (R = 0.95, *p* < 0.01), water temperature, Chl-a, and NH_3_-N. A previous study showed the dependence *of Pseudanabaena*, the main 2-MIB producer, on Chl-a, COD_Mn_, pH, and nitrogen levels [33]. It has generally been reported that high N and P levels support cyanobacterial growth [43]. A strong positive correlation was also found between *geo* copy number and geosmin concentration (R = 0.94, *p* < 0.01) (Figure 6B). However, in the environmental analysis, the only correlation was with water temperature (R = 0.46). 

Previous studies have analyzed the effects of temperature on odorant-producing cyanobacteria species, indicating that temperature is an important factor in odorant production [44,45].

Although environmental factors have been reported to be important for the growth of cyanobacteria and the consequent release of odorous compounds [33], evidence for a clear relationship between 2-MIB/geosmin production and environmental factors (light, temperature, nutrients, dissolved oxygen) has not yet been demonstrated [16]. Therefore, further research is required to clarify the relationship between environmental factors and odorant production. Furthermore, the presence of odorous substances in aquatic environments has a significant impact on water quality because the growth of certain odor-producing species may affect the structures and dynamics of other organisms in the ecosystem [5]. Therefore, further research on odor-causing blue-green algae and their ecological impact is required.

## 4. Conclusions

An accurate early analysis of 2-MIB/geosmin-producing cyanobacteria is essential for drinking water source management. In this study, we developed primers, considering the regional characteristics of cyanobacteria for qPCR analysis, to detect 2-MIB/geosmin-producing genes and applied them in the field to monitor two major odorants. Copy number analyses of *mic*/*geo*, 2-MIB/geosmin concentrations, and cyanobacterial cells were monitored using qPCR. Regression analysis was used to determine the relationship between gene copy number, odorant concentration, and blue–green algal cell abundance. We also investigated the differences in gene copy numbers between locations and the relationships between environmental factors.

The results showed that odorant concentration and gene copy number showed similar patterns of increase and decrease, and there was a strong linear relationship between *mic*/*geo* copy number determined with qPCR and 2-MIB/geosmin concentration. Among the various environmental factors, the gene copy number decreased from upstream to downstream, confirming that odorant genes are affected by water temperature. This study demonstrates the utility of a qPCR-based approach for monitoring 2-MIB/geosmin in predicting odor development. Future studies should move beyond DNA analysis and use RNA-based qPCR to analyze odorant expression in order to gain more detailed insights.

## Figures and Tables

**Table 1:** Primers designed for quantification of *mic* and *geo*.

Target (Gene)	Primer	Sequences, 5′→3′	Product Size (bp)	Tm (°C)
2-MIB	3909F	CAC CAG ATC TTT TCT TCG ATC	140	59
(*mic* ^1^)	4028R	AAT CTG TAG CAC CAT GTT GAC		
Geosmin	2014F	GCC CTT TTC GAC GAT TTC AA	150	59
(*geo* ^2^)	2163R	GGA AGC ACT ATT ACG CAA TTC AAA		

^1^ *mic* (*mibC*): *monoterpene cyclase gene*; ^2^ *geo* (*gsy1*): *putative geosmin synthase gene*.

## Data Availability

Not applicable.

## Supplementary Materials

The following supporting information can be downloaded from https://www.mdpi.com/article/10.3390/microorganisms11092332/s1. Table S1: Total cells of cyanobacteria, dominant species of cyanobacteria, cells of dominant species, and 2-MIB/geosmin concentrations at each sampling site.
