# Supplementary material for: qPCR-Based Monitoring of 2-Methylisoborneol/Geosmin-Producing Cyanobacteria in Drinking Water Reservoirs in South Korea
Source: Microorganisms. 2023 Sep 16;11(9):2332. doi: 10.3390/microorganisms11092332 (PMC10538080; doi:10.3390/microorganisms11092332)
Supplement: Supplementary file 1 [file microorganisms-11-02332-s001.zip › microorganisms-2520537-supplementary.pdf]

Table S1. Total cells of cyanobacteria, the dominant species of cyanobacteria, cells of the dominant species, and the 2-MIB/geosmin concentrations at each sampling site

Site	Date	Total cyanobacterial cells (cells ml^{-1})	Dominant sp. of cyanobacteria	Cells of dominant sp. (cells ml^{-1})	2-MIB (ng/L)	Geosmin (ng/L)
UA	Jul 13	4900	*Pseudanabaena* sp.	4900	28	9
	Jul 20	3000	*Pseudanabaena* sp.	3000	16	8
	Jul 27	7221	*Pseudanabaena* sp.	3800	31	14
	Aug 24	1200	*Pseudanabaena* sp.	1200	1	1
	Aug 31	500	*Pseudanabaena* sp.	500	2	2
	Sep 14	200	*Pseudanabaena* sp.	200	ND	1
CP	Jul 13	152	*Pseudanabaena* sp.	100	5	20
	Jul 20	237	*Pseudanabaena* sp.	200	5	7
	Jul 27	3093	*Phormidium* sp.	1600	21	12
	Aug 24	300	*Pseudanabaena* sp.	300	ND	1
	Aug 31	400	*Pseudanabaena* sp.	300	2	3
	Sep 28	600	*Pseudanabaena* sp.	600	1	1
SB	Jul 20	100	*Pseudanabaena* sp.	100	10	5
	Jul 27	2500	*Phormidium* sp.	2100	19	5
	Aug 24	60	*Microcystis aeruginosa*	60	1	1
	Aug 31	300	*Pseudanabaena* sp.	300	2	2
	Sep 14	200	*Pseudanabaena* sp.	200	ND	1
	Oct 19	10	*Pseudanabaena sp*	10	2	2
PD	Jul 6	112	*Anabaena circinalis*	112	1	29
	Jul 13	151	*Anabaena circinalis*	90	3	32
	Jul 27	120	*Merismopedia tenuissima*	120	13	6
	Aug 31	320	*Merismopedia tenuissima*	320	4	5
	Sep 21	2060	*Merismopedia tenuissima*	1250	1	1
